# Anti-Photoaging Effect of *Rhodiola rosea* Fermented by *Lactobacillus plantarum* on UVA-Damaged Fibroblasts

**DOI:** 10.3390/nu14112324

**Published:** 2022-06-01

**Authors:** Hao Fu, Yuzhi Zhang, Quan An, Dongdong Wang, Shiquan You, Dan Zhao, Jiachan Zhang, Changtao Wang, Meng Li

**Affiliations:** 1 Beijing Key Laboratory of Plant Resource Research and Development, College of Chemistry and Materials Engineering, Beijing Technology and Business University, Beijing 100048, China; 18811359600@163.com (H.F.); zhangyuzhi099@163.com (Y.Z.); wdd@btbu.edu.cn (D.W.); 13269182262@163.com (S.Y.); zhao_dan@btbu.edu.cn (D.Z.); xiao-chan8787@163.com (J.Z.); wangct@th.btbu.edu.cn (C.W.); 2 Institute of Cosmetic Regulatory Science, Beijing Technology and Business University, Beijing 100048, China; 3 Technical Research and Development Center, Yunnan Baiyao Group Co., Ltd., Kunming 650000, China; anquan@ynbyjk.com

**Keywords:** *Lactobacillus plantarum*, fermentation broth, *Rhodiola rosea*, anti-photoaging, UVA, human skin fibroblasts

## Abstract

UVA can cause oxidative stress and photoaging of cells. We established a UVA-induced oxidative stress model of human fibroblasts and focused on the antioxidant and anti-photoaging ability of *Lactobacillus plantarum* fermented *Rhodiola rosea*. Compared with the unfermented *Rhodiola rosea*, *Lactobacillus plantarum* fermented *Rhodiola rosea* has better DPPH free radical and hydroxyl free radical scavenging ability, significantly reduces the content of reactive oxygen species (ROS), and improves the antioxidant level. Further studies have shown that the *Lactobacillus plantarum* fermented *Rhodiola rosea* can activate the Nrf2/Keap1 signaling pathway and up-regulate heme oxygenase-1 (HO-1), NAD(P)H quinone dehydrogenase 1 (NQO1), catalase (CAT) and glutathione Peptide peroxidase (GSH-Px), and protect fibroblasts from oxidative stress caused by UVA. On the other hand, *Lactobacillus plantarum* fermented *Rhodiola rosea* significantly reduces the activity of metalloproteinases in the cell, thereby increasing the collagen and elastin in the cell, alleviating the photoaging caused by UVA. Finally, we concluded that the antioxidant capacity and anti-photoaging ability of *Lactobacillus plantarum* fermented *Rhodiola rosea* are better than that of unfermented *Rhodiola rosea*.

## 1. Introduction

*Rhodiola rosea* L. belongs to the plant family Crassulaceae. It is a traditional medicinal plant in China [1,2,3]. It is often used as a wine and medicinal preparation. Rhodiola is rich in active compounds, including salidroside, flavonoids, coumarin, organic acid compounds, etc., which have antioxidant, anti-inflammatory, anti-aging, and anti-fatigue effects [4,5,6]. Existing studies have found that Rhodiola after Alcaligenes piechaudii CC-ESB2 fermentation can increase the antioxidant activity and tyrosinase inhibitory activity [7]. Other studies have found that Rhodiola after the fermentation of Lactobacillus acidophilus KFRI 128 significantly reduces mice fatigue caused by strenuous exercise, and the effect is better than the unfermented *Rhodiola rosea* [8].

Human skin is often exposed to ultraviolet radiation from the sun. Current research shows that UVA (315–400 nm) is the main factor in photoaging. UVA damages DNA by causing strand breaks and oxidation of nucleic acids. DNA damage is one of the most serious effects of skin overexposure to UV radiation, and it plays a major role in the induction of photocarcinogenesis and is also directly involved in photoaging [9]. Studies have shown that compared with UVB, UVA will produce more oxidative stress [10]. UVA radiation to the dermal layer of the skin stimulates dermal cells to produce ROS, mainly manifested as the content of ROS increases. Reactive oxygen species participate in various complex activities in the cell, such as gene expression, protein production, and lipid oxidation [11,12,13]. ROS is also related to various pathologies such as inflammation, aging, and oxidation. Excessive reactive oxygen species will activate the nuclear factor red blood cell-related factor 2 (Nrf2) [14,15,16]. Nrf2 activated by the dissociation of Keap1 binds to antioxidant response elements and up-regulates the transcription of several different types of genes. There are many ways for cells to resist oxidative damage caused by ROS or directly reduce free radicals through a series of antioxidants. Defense mechanisms can be used to reduce oxidative stress caused by the excessive production of free radicals in cells. Enzyme defense systems include superoxide dismutase (SOD), catalase (CAT), and glutathione peroxidase (GPx), as well as other antioxidant enzymes to improve the effect [17,18]. Non-enzymatic defense systems include antioxidants, such as protecting cells from oxidative stress through vitamins E and C, glutathione (GSH), and various carotenoids and flavonoids [19].

The specific manifestations of aging are skin sagging, dryness, and wrinkles [20]. The structure and function of the extracellular matrix (ECM) have also changed. Type I collagen constitutes the basic framework and main components of ECM. Type III collagen is the main collagen for the elasticity of human skin with finer fibers. The tensile strength is low, so it is called “embryo” or “immature” collagen [21]. The degradation of collagen is affected by the content of matrix metalloproteinases (MMP). Therefore, it is generally believed that by promoting the synthesis of skin collagen and reducing the activity of MMPs, the skin aging process can be improved [22].

The purpose of this study is to prepare *Rhodiola rosea* fermentation broth by fermentation of *Lactobacillus plantarum*, with *Rhodiola rosea* water extract as a control, and to investigate the antioxidant mechanism of *Lactobacillus plantarum* fermented *Rhodiola rosea* by exploring the expression of the Nrf2/Keap-1 signaling pathway, and by exploring the effect on the collagen synthesis in HDF and the inhibitory effect on metal matrix protease to explore the anti-photoaging mechanism of *Lactobacillus plantarum* fermented *Rhodiola rosea*.

## 2. Results

### 2.1. Active Substance Content

First, the active substances in the *Lactobacillus plantarum* fermented *Rhodiola rosea* and unfermented *Rhodiola rosea*: polysaccharides, proteins, polyphenols, and flavonoids were determined. The experimental results are shown in Table 1. After fermentation by Lactobacillus plantarum, the polysaccharide and protein contents in the Lactobacillus Plantarum fermented *Rhodiola rosea* (FBOR) were significantly higher at the unfermented *Rhodiola rosea* (WEOR). However, the content of flavonoids did not increase significantly, indicating that the polysaccharide and protein content of *Rhodiola rosea* after being fermented by *Lactobacillus plantarum* would increase.

### 2.2. In Vitro Free Radical Scavenging Ability of WEOR and FBOR

**Figure 1:**
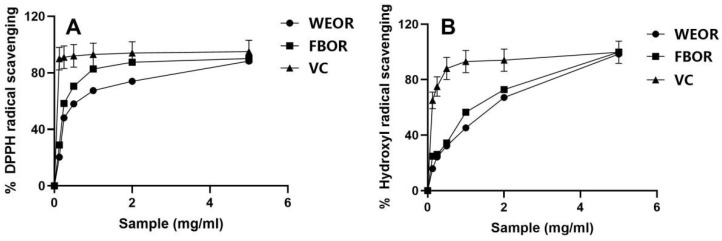
Different in vitro experiments were used to explore the antioxidant capacity of *Lactobacillus plantarum* fermented *Rhodiola rosea* and unfermented *Rhodiola rosea*. (**A**) DPPH free radical scavenging activity of lactobacillus *plantarum* fermented *Rhodiola rosea* and unfermented *Rhodiola rosea* at the same concentration. (**B**) The hydroxyl radical scavenging activity of *Lactobacillus plantarum* fermented *Rhodiola rosea* and unfermented *Rhodiola rosea* at the same concentration. Among them, Vitamin C (VC) is the positive control of the experiment.

Figure 1A shows the scavenging ability of WEOR and FBOR to DPPH free radicals. The half inhibitory concentration (IC50) values of WEOR and FBOR scavenging rate calculated by SPSS are 0.639% and 0.227%. The results showed that the DPPH free radical scavenging activity of the fermentation broth was significantly stronger than that of the water extract at the same concentration. Figure 1B shows the scavenging ability of WEOR and FBOR on hydroxyl radicals. The IC50 values of WEOR and FBOR are 0.837% and 0.409%. The results show that the scavenging ability of FBOR is stronger than that of WEOR at the same concentration. Therefore, Rhodiola fermented by *Lactobacillus plantarum* can significantly improve the free radical scavenging ability.

### 2.3. Cell Viability

**Figure 2:**
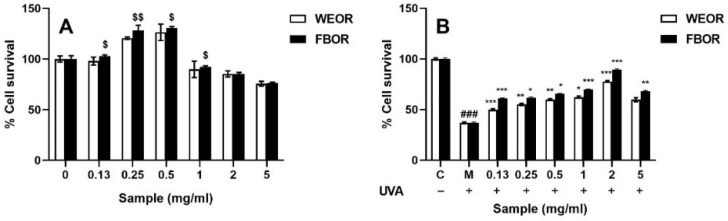
(**A**) Nontoxic concentrations of WEOR and FBOR-related cytotoxicity in HDF cells; (**B**) WEOR and FBOR prevent cytotoxicity in HDF cells exposed to UVA. (* *p* < 0.05, ** *p* < 0.01, *** *p* < 0.001, versus the UVB irradiation model group; ### *p* < 0.001, versus the control group. $ *p* < 0.05, $$ *p* < 0.01, the WEOR group versus the FBOR group. C: Control group; M: UVB irradiation model group).

The CCK-8 method was used to detect the cytotoxic effects of WEOR and FBOR on HDF cells. The culture dose of WEOR and FBOR affects the proliferation of HDF cells. As shown in Figure 2A, WEOR and FBOR show relatively low toxicity. When the volume fraction is less than 0.5 mg/mL, cell proliferation will occur. As shown in Figure 2B, the UVA-induced cytotoxicity of WEOR and FBOR was also measured. Within 24 h after UVA induction, different volume fractions of WEOR and FBOR can protect cells from UVA radiation. When the volume fraction is 2 mg/mL, the cell viability reaches its maximum. Therefore, 2 mg/mL was the optimal dose for the experiment.

### 2.4. Influence of WEOR and FBOR on Antioxidant Levels in Cells

**Figure 3:**
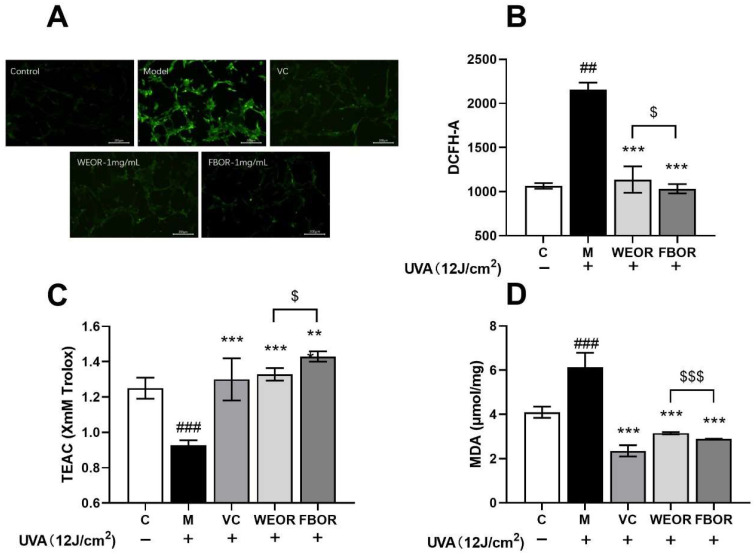
The content of ROS in HDF cells was detected by DCFH-DA staining. (**A**) The photomicrograph shows (10×) green fluorescence was enhanced in UVA-exposed HDF cells, and WEOR and FBOR pretreatment reduce the content of UVA-induced ROS. (**B**) The graph shows the fluorescence intensity of DCFH-DA in the cell. (**C**) The total antioxidant level in cells (ABTS); (**D**) Intracellular lipid oxidation level. (** *p* < 0.01, *** *p* < 0.001, versus the UVB irradiation model group; ## *p* < 0.01, ### *p* < 0.001, versus the control group. $ *p* < 0.05, $$$ *p* < 0.001, the WEOR group versus the FBOR group. C: Control group; M: UVB irradiation model group).

In this study, the content of ROS in HDF cells irradiated by UVA increased significantly (Figure 3). WEOR and FBOR pretreatment significantly inhibited the formation of intracellular ROS and reduced the intensity of green fluorescence. The microscope image showed that due to the formation of ROS in the UVA-irradiated cells, high green fluorescence was observed (Figure 3). After WEOR and FBOR pretreatment, the green fluorescence is weakened due to the reduction in intracellular ROS production. Excessive production of reactive oxygen species will reduce the level of antioxidants in the cells and, at the same time, destroy lipid membranes. This leads to lipid peroxidation in the cell, which, in turn, leads to oxidative stress. Therefore, we first detect the total antioxidant capacity in cells by detecting the level of ABTS free radicals (Figure 3C). It was observed that the total antioxidant levels in the cells were significantly reduced in HDF cells exposed to UVA. After WEOR and FBOR pretreatment, the antioxidant level in the cells was significantly increased, showing a certain dose-dependence, and then the intracellular malondialdehyde content was detected (Figure 3D). It can be seen that WEOR and FBOR effectively reduce the intracellular malondialdehyde and intracellular lipid content after UVA induction.

### 2.5. WEOR and FBOR Can Cause Nuclear Translocation of Nrf2 in Cells

**Figure 4:**
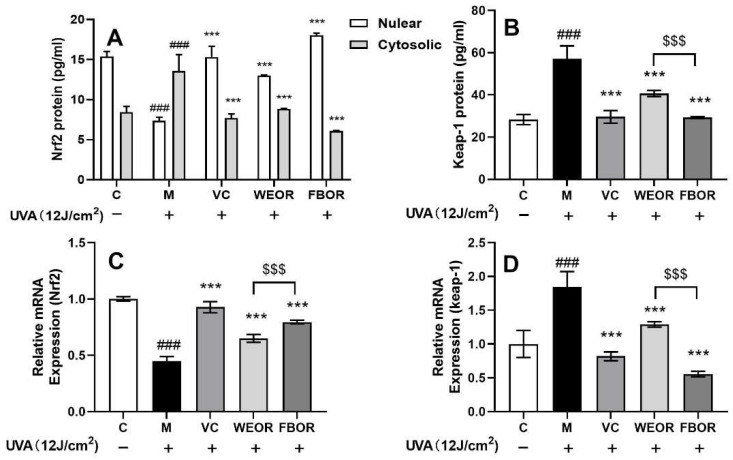
WEOR and FBOR activate the Nrf2-Keap-1 pathway: (**A**) Cytoplasmic Nrf2 protein content; Nuclear Nrf2 protein content; (**B**) Intracellular Keap-1 protein content; (**C**) Relative expression content of Nrf2 mRNA factor; (**D**) Relative expression content of Keap-1 mRNA factor. (*** *p* < 0.001, versus the UVB irradiation model group; ### *p* < 0.001, versus the control group. $$$ *p* < 0.001, the WEOR group versus the FBOR group. C: Control group; M: UVB irradiation model group).

We determined the effects of the nuclear translocation of Nrf2 mediated by WEOR and FBOR (Figure 4). ELISA data shows that after WEOR and FBOR pretreatment, the cytoplasmic cytosolic Nrf2 protein level in HDF cells decreased, while the nuclear Nrf2 protein level increased and the Keap-1 protein content decreased, and we also observed WEOR and FBOR treatment at the mRNA level. Later, the treatment up-regulated the level of Nrf2 in HDF cells in a dose-dependent manner and down-regulated Keap-1. Therefore, we can conclude that after HDF cells exposed to UVA are pretreated with WEOR and FBOR, the Nrf2-Keap-1 complex is dissociated in the cytoplasm, and Keap-1 protein is degraded. This is conducive to the translocation of Nrf2 to the nucleus to mediate its downstream antioxidant effects.

### 2.6. Influence of WEOR and FBOR on Antioxidant Levels in Cells

**Figure 5:**
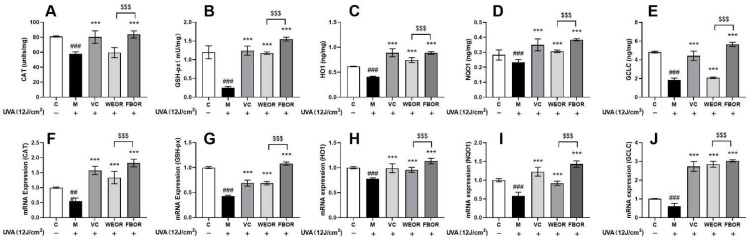
The effect of WEOR and FBOR on the level of antioxidant enzyme activity in HDF after UVA irradiation: (**A**,**F**) CAT activity and mRNA relative expression; (**B**,**G**) GSH-Px activity and mRNA relative expression; (**C**,**H**) HO-1 activity and mRNA relative expression; (**D**,**I**) NQO1 activity and mRNA relative expression; (**E**,**J**) GCLC activity and mRNA relative expression. (*** *p* < 0.001, versus the UVB irradiation model group; ## *p* < 0.01, ### *p* < 0.001, versus the control group. $$$ *p* < 0.001, the WEOR group versus the FBOR group. C: Control group; M: UVB irradiation model group).

The expression of CAT, GSH-Px, GCLC, HO-1, and NQO1 proteins is up-regulated by the transcriptional activation of Nrf2 in HDF cells (Figure 5). The induction of CAT, GSH-Px, GCLC, HO-1, and NQO1 antioxidant genes is the main mechanism for scavenging ROS to protect cells from oxidative damage. This effect is mediated through the nuclear translocation of Nrf2. Therefore, we found through ELISA and RT-PCR analysis that WEOR and FBOR treatment significantly increased the levels of CAT, GSH-Px, GCLC, HO-1, and NQO1 proteins. From this, we conclude that WEOR and FBOR mediate the nuclear localization of Nrf2 transcriptional activation and further promote the induction of GCLC, HO-1, and NQO1 proteins in HDF cells. Moreover, compared with WEOR, FBOR reduces the oxidation level in cells more effectively.

### 2.7. Effects of WEOR and FBOR on the Activity of MMPs in Cells Irradiated by UVA

**Figure 6:**
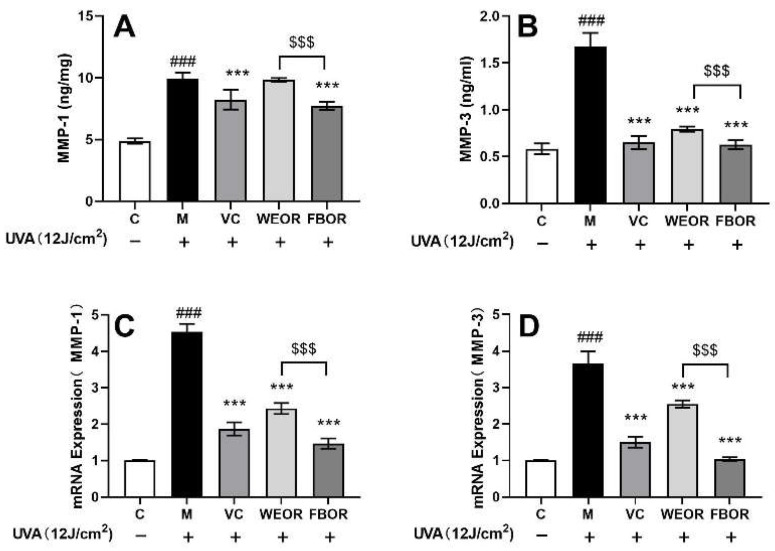
Effects of WEOR and FBOR on the activity and expression of MMPs in fibroblasts: (**A**) MMP-1 protein content; (**B**) MMP-3 protein content; (**C**) MMP-1 mRNA expression level; (**D**) MMP-3 mRNA expression level. (*** *p* < 0.001, versus the UVB irradiation model group; ### *p* < 0.001, versus the control group. $$$ *p* < 0.001, the WEOR group versus the FBOR group. C: Control group; M: UVB irradiation model group).

This study examined the ability of fermented (FBOR) and non-fermented (WEOR) preparations to inhibit the activities of MMP-1 and MMP-3. WEOR had no significant inhibitory effect on MMP-1 protein (Figure 6A) and a significant inhibitory effect on MMP-3 (Figure 6B), but in terms of the mRNA level (Figure 6C,D), WEOR can significantly down-regulate the expression of MMP-1 and MMP-3. At the same level, FBOR inhibited the production of MMP-1 and MMP-3 and down-regulated the expression of MMP-1 and MMP-3 mRNA. The higher the concentration, the stronger the inhibitory effect. The inhibitory effect of MMP-3 is significantly different, so Rhodiola, after fermentation, can more effectively inhibit the degradation of intracellular collagen.

### 2.8. Influence of WEOR and FBOR on Collagen and Elastin Activity

**Figure 7:**
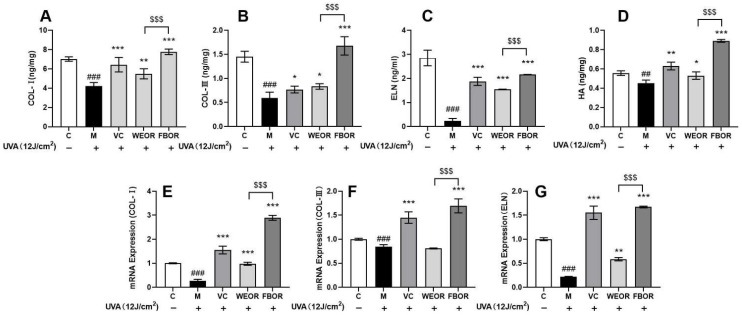
The effects of WEOR and FBOR on intracellular collagen and elastin content: (**A**) Type I collagen content; (**B**) Type 3 collagen content; (**C**) Elastin content; (**D**) Hyaluronic acid content; (**E**) Relative expression of type I collagen mRNA; (**F**) Relative expression of type III collagen mRNA; (**G**) Relative expression of elastin mRNA. (* *p* < 0.05, ** *p* < 0.01, *** *p* < 0.001, versus the UVB irradiation model group; ## *p* < 0.01, ### *p* < 0.001, versus the control group. $$$ *p* < 0.001, the WEOR group versus the FBOR group. C: Control group; M: UVB irradiation model group).

This article further tested the effects of WEOR and FBOR on the content of collagen, elastin, and hyaluronic acid. ELISA detects intracellular protein levels, and RT-PCR detects intracellular mRNA levels. It can be seen from Figure 7 that after UVA irradiation, Col-1, Col-3, and ELN in the cells are degraded. After pretreatment with WEOR and FBOR, the content of Col-1, Col-3, ELN, and hyaluronic acid in the cells increased significantly, and the expression of the genes Col-1, Col-3, and ELN was up-regulated. Therefore, it can be concluded that WEOR and FBOR can effectively increase the content of collagen and elastin in cells in a dose-dependent manner, and the effect of Rhodiola fermentation is better.

## 3. Discussion

The clinical application of Chinese medicine has a long history. Driven by consumers’ growing interest in and demand for natural products, the application of Chinese herbal medicine in the development of skincare cosmetics has aroused great interest and attention [23,24,25]. Currently, various Chinese herbal medicines are used in skincare cosmetics, and it is claimed that they can improve the physical appearance of aging skin [26,27]. Some studies have claimed that fermentation is an indispensable traditional process for improving the efficacy of Chinese herbal medicines or reducing their adverse reactions. Studies have proven that Rhodiola can prevent oxidative stress in cells and possesses antioxidant and anti-inflammatory effects [28,29,30]. After fermentation of Rhodiola by Lactobacillus plantarum, we detected that the content of active substances in Rhodiola rose significantly, including polysaccharides, polyphenols, and flavonoids, but the specific changes of active substances in the fermentation process need to be further studied. At present, studies have shown that salidroside, flavonoids, and phenols in Rhodiola have excellent free radical scavenging activity [31]. Therefore, we speculate that the effect of *Rhodiola rosea* fermented liquid is better than that of unfermented *Rhodiola rosea* due to the modification and optimization of the active substances in *Rhodiola rosea* by the metabolites of Lactobacillus plantarum, and the specific mechanism of action needs to be further studied [32]. All the results showed that fermented Rhodiola has a better effect on cells. 

Exposure to UVA causes direct damage to skin cells through an inflammatory reaction and indirectly through induced oxidative stress. This initiates the peroxidation of polyunsaturated fatty acids (PUFA) in the skin membrane and the formation of a DNA adduct, 8-hydroxy-2’-deoxyguanosine (8-OHdG), which is the most numerous and highly mutagenic factor considered a reliable marker for oxidative DNA damage [33,34]. Therefore, UVA radiation triggers the accumulation of intracellular ROS levels in different cell types. The increase in ROS levels leads to oxidative stress in skin cells, which is believed to be a precursor to premature skin aging [35,36]. In this study, we first detected an increase in ROS levels in HDF cells after UVA irradiation. However, the ROS levels decreased after pretreatment with WEOR and FBOR. All these observations are evidence of the antioxidant and anti-aging abilities of these two mixtures in skin cells and compared to WEOR, FBOR reduces the level of intracellular ROS more significantly.

The transcription factor Nrf2 (NF-E2 related factor 2) is the main regulator of cellular antioxidant response element activation, which induces the expression of antioxidant enzymes in the cell [37,38]. The activity of Nrf2 is partly controlled by its cytoplasm-related protein Keap-1. The separation of Keap1 and Nrf2 determines cell homeostasis [39]. Our results show that after pretreatment with WEOR and FBOR, the cytoplasmic Nrf2 content decreases, while the nuclear Nrf2 increases, which means that WEOR and FBOR can dissociate and nuclear translocate Nrf2-Keap-1 in the cytoplasm to protect cells from oxidation stress.

Antioxidant genes HO-1, GCLC, SOD, CAT, and GSH-Px are the downstream genes of the Nrf2-Keap-1 pathway, and their main role is to remove ROS to protect cells from oxidative damage [39,40]. The research results show that after WEOR and FBOR treatment, the expression of antioxidant enzyme mRNA was up-regulated, which increased the antioxidant level of cells, and it can be seen from the Figure that the antioxidant capacity of FBOR is better than WEOR.

The increase in ROS content stimulates the synthesis of MMP-1. MMP-1 can degrade collagen in the skin, especially type I and type III collagen, and other types of MMP further degrade collagen fragments, so the increase in intracellular MMP-1 leads to collagen fragments, accumulation of MMP-1 and MMP-9 mRNA, and damage to the structure and function of the extracellular matrix (ECM) [41,42]. After research, we found that the expression of MMP-1 and MMP-9 mRNA was up-regulated in cells after UVA irradiation. After FBOR pretreatment, it down-regulates the expression of MMP-1 and MMP-9 mRNA. Compared with WEOR, FBOR has a greater down-regulation effect on MMP-1 and MMP-9. Therefore, the fermented Rhodiola can more effectively inhibit the activity of MMP-1 and MMP-9.

The main component of the extracellular matrix is collagen, and the collagen in the skin is mainly type I and type III collagen. Among them, type I collagen is mature collagen with strong tensile strength, accounting for about 85% of the total collagen [43], while type III collagen is immature collagen with low tensile strength, and its content increases with age. It is greatly reduced, about 15% of total collagen [44,45]. After our research, we confirmed that WEOR and FBOR up-regulated the expression of COLI and COLIII mRNA. We also confirmed that WEOR and FBOR can increase type I and type III collagen in the skin, which is effective in reducing the photoaging of cells.

## 4. Experimental

### 4.1. Materials

Human skin fibroblasts (HSF), China Institute of Inspection and Quarantine; BCA Protein Quantification Kit, Biorigin; Phosphate-Buffered Saline (PBS), Fetal Bovine Serum, Beyotime; ROS, MDA, SOD, GSH-Px, COL-1, MMP-1, etc., Assay kits, Nanjing Jiancheng Institute of Bioengineering(Nanjing, China); FM media, Corning Inc. (New York, NY, USA); pipette, 96-well plate, Thermo Fisher Scientific; microplate reader, UVA light tube, Shenzhen Guanhon-gruit Technology Co., Ltd. (Shenzhen, China); BCA Protein Assay Kit, Biorigin (Beijing, China) Inc. qPCR related kit, Tiangen Biotech (Beijing, China) Co., Ltd.

### 4.2. Lactobacillus Plantarum Fermented Rhodiola rosea

Rhodiola was purchased from Beijing Tongrentang Co., Ltd. (Beijing, China), dried, mechanically crushed, and passed through a 40-mesh sieve. In total, 50 g of Rhodiola powder was mixed into 1 L of distilled water, heated and stirred at 70 °C for 12 h, cooled at room temperature, centrifuged at 4800 r/min for 30 min, and sterilized at 121 °C for 20 min. The supernatant was obtained for fermentation. The supernatant was the unfermented *Rhodiola rosea* (WEOR). The *Lactobacillus Plantarum* was purchased from the China Center of Industrial Culture Collection, the strain number is CICC20261, and 3% (*v*/*v*) *Lactobacillus plantarum* was added. It is used for the fermentation of Rhodiola; the fermentation temperature was 37 °C, the fermentation time was 24 h, the fermentation broth after fermentation was sterilized at 121 °C for 20 min, and the supernatant was collected to obtain the *Lactobacillus Plantarum* fermented *Rhodiola rosea* (FBOR). All fermentation broth experiments were repeated three times to ensure the stability of the fermentation broth [7].

### 4.3. Measurement of Polysaccharides, Flavonoids, and Protein Content

We used phenol-sulfuric acid to determine the polysaccharide content and D-glucose as the standard. The content of flavonoids was determined by nitrite colorimetry, and rutin was used as a standard. The BCA kit was used to determine the protein content. Please refer to the manufacturer’s instructions for specific steps.

### 4.4. Antioxidant Activity Analysis

For the method used to study the DPPH free radical scavenging activity of *Rhodiola rosea* fermentation broth, please refer to the previously published method. In general, first, ethanol was used to configure the DPPH solution, the DPPH concentration was 0.12 mg/mL, and then different concentrations (0.1–5 mg/mL) samples were configured. In total, 1 mL sample and 1 mL DPPH solution were mixed, incubated in the dark at room temperature for 30 min, and finally the absorbance was measured at 517 nm. Taking VC as a positive control, the DPPH free radical scavenging rating formula is as follows:

DPPH free radical scavenging rate % = (A2 + A3 − A1)/A2 × 100%

A1 is the absorbance of the ethanol DPPH solution and the sample; A2 is the absorbance of the DPPH ethanol solution as a blank control; A3 is the absorbance of the sample in the ethanol solution.

The determination of the hydroxyl radical scavenging ability of Rhodiola fermentation broth is used after modification according to the previous research method. The specific operation is as follows: configure samples of different concentrations (0.1–5 mg/mL), add 2 mL 6 mmol/L to the test tube sequentially FeSO_4_, 2 mL samples of different dilution multiples, 2 mL 6 mmol/L H_2_O_2_ (where H_2_O_2_ is added last, that is to start the entire reaction), shake well, and stand still at room temperature for 10 min. Then, add 2 mL 6 mmol/L salicylic acid, shake well, heat it in a 37 °C water bath for 30 min, take it out, and measure its absorbance Ai; add 2 mL 6 mmol/L FeSO_4_ to the test tube successively, 2 mL of the sample to be tested with different dilution multiples, 2 mL 6 mmol/L H_2_O_2_, shake well, stand at room temperature for 10 min, then add 2 mL deionized water, shake well, heat in a 37 °C water bath for 30 min, take it out, measure the absorbance Aj; add 2 mL 6 mmol/L FeSO_4_, 2 mL deionized water, 2 mL to the test tube in turn 6 mmol/L H_2_O_2_, shake well, stand still at room temperature for 10 min, then add 2 mL 6 mmol/L salicylic acid, shake well, heat in a 37 °C water bath for 30 min, take it out, and measure its absorbance A0.

Clearance rate (%) = [(A0 + Aj) − Ai]/A0 × 100%

### 4.5. Cell Culture

Human immortalized fibroblasts (HDF) (Shanghai Institute of Biological Sciences, Shanghai, China) were cultured in DMEM from Gibco, then supplemented with 10% FBS, 1% fibroblast growth additive, and 2% penicillin-streptomycin, all of which were from Gibco. They were then cultured in a humidified atmosphere at 37 °C and 5% CO_2_, and the medium was changed every two days.

### 4.6. Irradiation Procedure

HDF cells were seeded in a 96-well plate at a density of 8 × 103 cells/well. The cells were covered with a layer of phosphate-buffered saline (PBS) and irradiated under UVA for 3 h. The total amount of UVA radiation given was 12 J/cm^2^. After the irradiation, the cells were cultured in a constant temperature and humidity incubator at 37 °C and 5% CO_2_ for 24 h, then the CCK8 method was used to detect cell viability.

### 4.7. Assay of Cell Viability

The standard CCK8 colorimetric method was used to evaluate the toxicity of the Rhodiola fermentation broth. The cells were seeded in a 96-well plate (100 μL/well) at a density of 8 × 103/well. After culturing for 8–12 h, the *Rhodiola rosea* fermentation broth was added to a 96-well plate with a sample concentration of 0.3125% to 40%. After 24 h of incubation, the cells were cultured at 37 °C in 10 μL of CCK8 medium. After 2 h, the optical density was then measured at 450 nm. The cell survival rate is expressed as the proportion of living cells in the presence of the extract compared with the blank group; “blank” refers to cells that have not been treated with the extract.

Next, according to the above method, 12 h after treating the sample, the HDF cells were irradiated with 12 J/cm^2^ UVA to determine the photoprotection effects of the Rhodiola fermentation broth on the cells.

### 4.8. ROS

The cell culture is the same as 2.5; the cells were treated with the Rhodiola fermentation broth, then cultured at 37 °C and 5% CO_2_ for 4 h after irradiation with UVA. For cell precipitation, refer to the instructions of the ROS Detection Kit. Fluorescence detection was then performed (with three replicate holes set for each sample) [22].

### 4.9. Antioxidants and Lipid Peroxidation Levels

The cell culture is the same as 4.5; they were incubated with or without TFPS for 24 h, then irradiated with UVA. After the cells were oxidatively stressed, they were washed twice with PBS and lysed, and the lysate was collected for subsequent testing. The corresponding assay kit purchased from Shanghai Beyotime Biotechnology(Shanghai, China) was then used to measure the content of ABTS and MDA according to the instructions provided in the kit [7,23,24,25].

### 4.10. Enzyme-Linked Immunosorbent Assay

Enzyme-linked immunosorbent assay (ELISA) was used to detect intracellular proteins (Nrf2, Keap-1, MMP-1, MMP-3, HO-1, NQO1, GCLC, COL-1, COL-3, ELN). The detection protocol was carried out in strict accordance with the instructions of the kit manufacturer.

### 4.11. RT-PCR

After treatment with the Rhodiola fermentation broth and UVA irradiation, the total RNA was extracted from the HDF cells using a trizol RNA extraction reagent (trizol, Sigma-Aldrich (Shanghai, China) Trading Co, Ltd.). Before that, an experiment was performed to ensure the purity of the extracted mRNA using Nanotrop (Thermo Fisher Scientific, Waltham, MA, USA), and the cDNA and β-actin genes were synthesized as an internal control according to the manufacturer’s instructions. The PCR conditions were as follows: 94 °C, 5 s; 60 °C, 15 s; 72 °C, 10 s; 94 °C, 5 s; 72 °C, 10 s; 40 cycles in duration. The primer sequence list is shown in Table 2 [26].

### 4.12. Data Analysis

All measurements and data were carried out in three independent experiments, all values are expressed as mean ± standard deviation (SD), and all statistical calculations using the IBM SPSS Statistics 22 software program (Chicago, IL, USA). *p* < 0.05 is considered to indicate statistically significant differences. 

## 5. Conclusions

This study provides strong evidence that FBOR protects human skin fibroblasts from oxidative stress caused by UVA irradiation. We have proved that FBOR can increase the enzyme activity of antioxidant enzymes in the cell, delay aging by reducing the activity of MMPs, increasing the content of collagen and elastin, and compared with WEOR, we found that we believe that fermented Rhodiola has better application prospects for internal and external skincare. However, further research is needed on the active substances that affect the skin in FBOR.

## Figures and Tables

**Table 1:** Contents of polysaccharides, proteins, total phenols, and flavonoids in WEOR and FBOR.

Concentration (mg/mL)	WEOR	FBOR
Polysaccharides	6.03 ± 0.05 ^a^	7.58 ± 0.25 ^b^
Proteins	2.92 ± 0.12 ^c^	4.11 ± 0.02 ^d^
Total phenols	0.17 ± 0.01 ^e^	0.25 ± 0.03 ^f^
Flavonoids	0.006 ± 0.0001 ^g^	0.007 ± 0.0003 ^g^

Values are expressed as Mean ± SD, and different letters in the same column indicate significant differences in data (*p* < 0.05).

**Table 2:** The primer sequences of key genes.

Primer Name		Primer Sequences (5′–3′)
Keap1	F	GGAGGCGGAGCCCGA
	R	GATGCCCTCAATGGACACCA
Nrf2	F	CAACTCAGCACCTTGTATC
	R	TTCTTAGTATCTGGCTTCTT
HO1	F	CAAGCGCTATGTTCAGCGAC
	R	GCTTGAACTTGGTGGCACTG
NQO1	F	CAGCCAATCAGCGTTCGGTA
	R	CTTCATGGCGTAGTTGAATGATGTC
CAT	F	CCTTCGACCCAAGCAA
	R	CGATGGCGGTGAGTGT
SOD	F	TGGAGATAATACAGCAGGCT
	R	AGTCACATTGCCCAAGTCTC
GSH-Px	F	AGAAGTGCGAGGTGAACGGT
	R	CCCACCAGGAACTTCTCAAA
MMP-1	F	GCA TATCGATGCTGCTCTTTC
	R	GATAACCTGGATCCATAGATCGTT
MMP-3	F	CAA AACATATTTCTTTGTAGAGGACAA
	R	TTCAGCTATTTGCTTGGGAAA
COL I	F	GTGCTAAAGGTGCCAATGGT
	R	GTGGGGAATGGCAAGCAAAA
COL III	F	CCAGGAGCTAACGGTCTCAG
	R	CAGGGTTTCCATCTCTTCCA
β-actin	F	TGGCACCCAGCACAATGAA
	R	CTAAGTCATAGTCCGCCTAGAAGC

F: forward primer; R: reverse primer.

## Data Availability

The data are available from the corresponding author upon reasonable request.
